# An Investigation of *Culicoides* (Diptera: Ceratopogonidae) as Potential Vectors of Medically and Veterinary Important Arboviruses in South Africa

**DOI:** 10.3390/v13101978

**Published:** 2021-10-01

**Authors:** Jumari Snyman, Gert J. Venter, Marietjie Venter

**Affiliations:** 1 Centre for Viral Zoonoses, Department Medical Virology, University of Pretoria, Pretoria 0001, South Africa; jumari.steyn@gmail.com; 2 HIV Pathogenesis Programme, The Doris Duke Medical Research Institute, University of KwaZulu-Natal, Durban 4001, South Africa; 3 Agricultural Research Council, Onderstepoort Veterinary Research, Pretoriat 0110, South Africa; venterjgert@gmail.com

**Keywords:** alphaviruses, flaviviruses, orthobunyviruses, *Rhabdoviridae*, Shuni virus, wildlife

## Abstract

*Culicoides*-borne viruses such as bluetongue, African horse sickness, and Schmallenberg virus cause major economic burdens due to animal outbreaks in Africa and their emergence in Europe and Asia. However, little is known about the role of *Culicoides* as vectors for zoonotic arboviruses. In this study, we identify both veterinary and zoonotic arboviruses in pools of *Culicoides* biting midges in South Africa, during 2012–2017. Midges were collected at six surveillance sites in three provinces and screened for *Alphavirs*, *Flavivirus*, *Orthobunyavirus*, and *Phlebovirus* genera; equine encephalosis virus (EEV); and *Rhaboviridae,* by reverse transcription polymerase chain reaction. In total, 66/331 (minimum infection rate (MIR) = 0.4) pools tested positive for one or more arbovirus. Orthobunyaviruses, including Shuni virus (MIR = 0.1) and EEV (MIR = 0.2) were more readily detected, while only 2/66 (MIR = 0.1) Middelburg virus and 4/66 unknown *Rhabdoviridae* viruses (MIR = 0.0) were detected. This study suggests *Culicoides* as potential vectors of both veterinary and zoonotic arboviruses detected in disease outbreaks in Africa, which may contribute to the emergence of these viruses to new regions.

## 1. Introduction

*Culicoides* midges have been implicated in the transmission of various pathogens of medical and veterinary importance [1]. The unprecedented spread of bluetongue virus (BTV) (*Reoviridae*: *Orbivirus*) [2] followed by the emergence of Schmallenberg virus (SBV) (*Peribunyaviridae*: *Orthobunyavirus*) into northern Europe [3] and the high incidence of African horse sickness in South Africa [4] highlights the importance of *Culicoides* midges (Diptera: Ceratopogonidae) as arbovirus vectors in establishing epidemics in animals. To date, over 50 arboviruses have been isolated from *Culicoides*, 45% of which have not been detected in other arthropods [1]. A recent study demonstrated human feeding to be as high as 11% in field collected *Culicoides* species, and as yet, little information exists on the role of *Culicoides* as vectors for zoonotic pathogens [5].

Biting midges have been implicated as the primary vectors of SBV and Oropouche virus (OROV) (*Peribunyaviridae*: *Orthobunyavirus*) which are responsible for congenital deformities in sheep and cattle and from mild febrile illness to severe disease in humans [3,6]. Arboviruses with zoonotic potential such as West Nile virus (WNV) (*Flaviviridae*: *Flavivirus*) [7], SHUV (*Peribunyaviridae*: *Orthobunyavirus*) [8], Dugbe virus (*Nairoviridae*: *Nairovirus*) [8], and Rift Valley fever virus (RVFV) (*Phenuiviridae*: *Phlebovirus*) [9] have also been isolated or detected in *Culicoides* pools, although the role of midges in the epidemiology of these viruses is unknown.

In this study, we screened *Culicoides* midges collected in three provinces in South Africa to identify zoonotic arboviruses that may contribute to disease in animals and humans in urban settings as well as at the human/livestock/wildlife interface.

## 2. Materials and Methods

Vector surveillance sites (Table 1) were selected based on previous outbreaks of zoonotic arboviruses in animal populations in South Africa [10,11,12,13,14,15]. Mpumalanga and Limpopo province are primarily wildlife regions and include various national parks including Kruger National Park (KNP), Marakele Nature Reserve, and Lapalala Wilderness. The northeastern corner of Bushbuckridge, known as Mnisi, which is a rural community, forms a human–animal interface with KNP. Gauteng is an urban area with numerous horse farms. Bluetongue and African horse sickness virus (AHSV) are also endemic to these regions. *Culicoides* midges were collected monthly from September 2012 to May 2017, at six sites in the three provinces, for two consecutive nights, using 220 V Onderstepoort light traps, as previously described [5,16]. Subsamples (10% of pooled sample) from various collections were morphologically identified to the species level to establish species composition at the surveillance sites (Table S1) [17].

*Culicoides* midges were separated from other insects using a light microscope and pooled (100 ≤ *n* ≤ 500), according to site and month, and homogenized using a TissueLyser™ (QIAGEN, Valencia, CA, USA). Following maceration, RNA was extracted using the QIAamp^®^ Viral RNA mini kit (QIAGEN), according to the manufacturer’s instructions. During the summer months, when insect numbers were high, testing was limited to only two randomly selected pools per site per month. Whereas during winter months, insect numbers declined, therefore, only one pool was available and tested and, in some cases, no insects were collected, and no pools tested (*n* < 100). Pool sizes were, therefore, determined based on the number of midges collected per site, per month, with a maximum of 500 and a minimum of 100 midges per pool.

Various published reverse transcription polymerase chain reactions (RT-PCRs) were used to screen pools for arboviruses in the genera *Alphavirus* [18], *Flavivirus*, [19] *Orthovirus* [20], and *Phlebovirus* [20], as well as equine encephalosis virus (EEV) [14] and *Rhabdoviridae* [21]. All orthobunyavirus PCR positives were followed up with a One-Step *Bunyaviridae* RT-PCR. The One Step *Bunyaviridae* conventional RTPCR was used to obtain a > 500 nt gene regions for known orthobunyavirus positives. SuperScript^®^ III/Platinum^®^ Taq Mix (ThermoFisher Scientific, MA, USA) was used in combination with 20 pmol each of BUN 1 and BUN 2 primers [22] in a 50 µL reaction. These primers amplify a 550 base pair fragment of the N gene of the S segment of orthobunyaviruses. Cycling conditions were as follow: 60 °C for 1 min, 50 °C for 30 min, 94 °C for 15 min, followed by 35 cycles of 94 °C for 45 s, 50 °C for 45 s, and 72 °C for 45 s, and a final extension of 10 min at 72 °C [12]. All SHUV positives were followed up with a SHUV M segment PCR (Appendix A). Good laboratory practice with separated pre and post PCR areas, as well as the inclusion of negative and positive controls, were used in all assays. PCR positive results were confirmed by sequencing at Inqaba Biotec (Pretoria, South Africa) and sequence data were analysed and trimmed using CLC Main Workbench version 8.0.1 (https://www.qiagenbioinformatics.com (accessed on 19 September 2019)) and MEGA 6.06 software (https://www.megasoftware.net (accessed on 20 September 2019)). Multiple sequence alignments were compiled using the online version of MAFFT version 7 software (http://mafft.cbrc.jp (accessed on 19 September 2019)). All assembled sequences were compared to the National Center for Biotechnology Information (NCBI) using the Basic Local Alignment Search Tool (BLAST) (http://www.ncbi.nlm.nih.gov/BLAST/ (accessed on 19 September 2019)). Sequences were also compared to positive controls to exclude false positives. The sequences >300 nucleotides (nt) were submitted to GenBank (Accession numbers MN270991–MN271022, Table S2). Maximum likelihood analyses were conducted on all the datasets in RaxML [23], starting with a random tree selection and estimated models. The bootstrap support values were calculated using the autoMRE bootstopping criterion in RaxML [24].

Statistical analyses were done in Epi Info version 7.2.0.1 (https://www.cdc.gov/epiinfo/index.html (accessed on 20 October 2019)) using Fisher’s exact test with a 95% confidence interval (CI). The minimum infection rate (MIR) was calculated, using PooledInfRate (www.cdc.gov/ncidod/dvbid/westnile/software.htm (accessed on 10 July 2021)), per location as: ((number of positive pools/total specimens tested) × 1000). The MIR uses the assumption that a positive pool contains at least one infected specimen [25].

## 3. Results

In total, more than 2,223,296 specimens of *Culicoides* were collected at six sites in 532 collections (mean ($\bar{x}$) = 4179) (Table 1). A total of 25 different *Culicoides* species were morphologically identified from 418 individuals (Table S1). From this, *Culicoides imicola* was the predominant species and present at all six sites. Seasonal trends could be observed with an increase in *Culicoides* numbers during summer and autumn months (January to May) with peak activity in February and a decrease in numbers during winter months (June to August), although never zero. In total, 66/331 pools tested positive for one or more arbovirus, resulting in a MIR of 0.4 (95% confidence interval (CI) 0.0–0.5) (Table 2). The highest MIR was recorded in Lapalala Wilderness (MIR = 0.8, 95% CI 0.5–1.2) and Marakele National Park (MIR = 0.5, 95% CI 0.2–0.6), both in Limpopo province (Table 2).

Orthobunyaviruses (33/331, MIR = 0.2, 95% CI 0.1–0.3) and EEV (30/331, MIR = 0.2, 95% CI 0.1–0.2) were equally abundant (*p* = 0.9). The orthobunyaviruses were further identified using phylogenetic analyses described below. Shuni virus (SHUV) was the predominant orthobunyavirus (18/33, MIR = 0.1, 95% CI 0.1–0.2) detected, while 15 other orthobunyaviruses (15/33, MIR = 0.1, 95% CI 0.0–0.1) were also identified (Table 2). Two Middelburg virus (MIDV) positives were the only alphaviruses detected (MIR = 0.1, 95% CI 0.0–0.4). The flavivirus generic PCR detected 4/331 (MIR = 0.2, 95% CI 0.0–0.3) unclassified *Rhabdoviridae* viruses which was confirmed with a *Rhabdoviridae* PCR. No viruses from the *Flavivirus* or *Phlebovirus* genera were detected. Co-infections were detected in pools from Lapalala, LAP775_17 and MAR057.2_13 (both SHUV/EEV), and Boschkop; GAU093_14 (Sabo virus/EEV) (Table 2).

### Sequencing and Phylogenetic Analysis of Positive Pools

**Figure 1:**
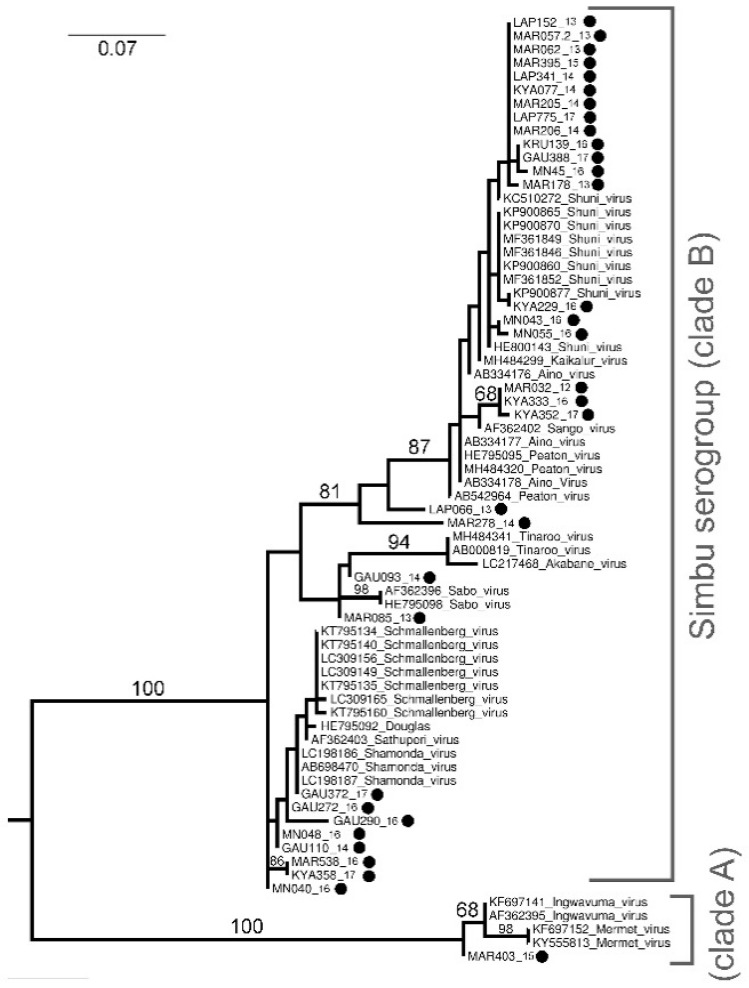
Maximum likelihood phylogram of the partial (nucleotides = 152, 70 taxa) *Orthobunyavirus* S segment. Bootstrap support (<60 omitted) calculated using autoMRE function invoked in RAxML (GTR + G model). Phylogram rooted at midpoint. Filled black circles indicate new sequences for viruses detected in this study.

**Figure 2:**
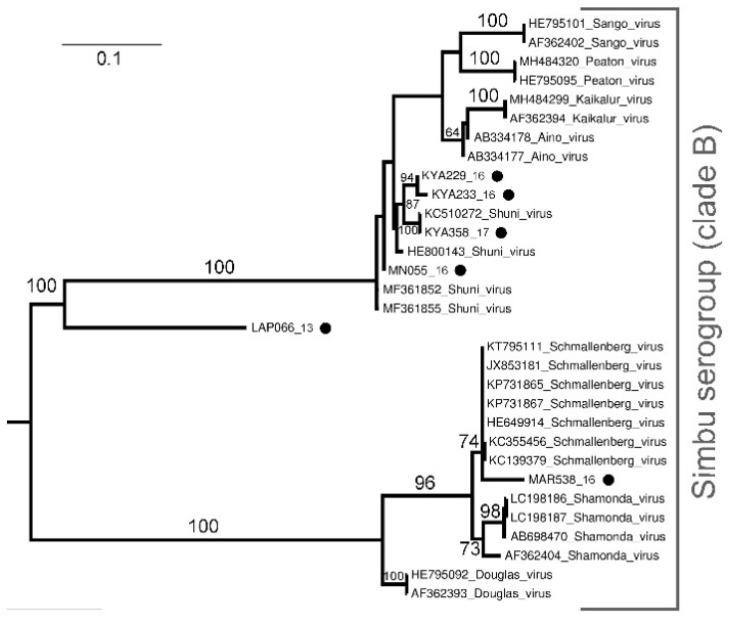
*Orthobunyavirus* phylogram of the partial (nucleotides = 503, 32 taxa) S segment. Bootstrap support (<60 omitted) calculated using autoMRE function invoked in RAxML (GTR + G model). Phylogram rooted at midpoint. Filled black circles indicate new sequences for viruses detected in this study.

*Orthobunyavirus* phylogenetic analysis based on a 152 nt sequence of the S segment identified two major clades of the Simbu serogroup, clade A (bootstrap (bs) = 100) and clade B (bs = 100) (Figure 1). Of these, 16 samples clustered in clade B and were closest to SHUV based on BLAST, p-distance analysis (100% nt similarity) (not shown) and phylogenetic clustering, although significant bootstrap values could not separate them from the other closely related members of this cluster (Figure 1). Further, 16 other *Orthobunyavirus* positives were also identified within clade A and B. These viruses include Shamonda virus (SHAV) (*n* = 5), Sabo virus (SABV) (*n* = 2), Sango virus (SANV) (*n* = 3), Schmallenberg virus (SBV) (*n* = 2), Satuperi virus (SATV) (*n* = 1), Ingwavuma virus (INGV) (*n* = 1), and two unknown viruses (Figure 1 and Figure 2). Sample MAR403_15 has a nucleotide identity of 98.1% (not shown) to INGV and confidently clusters into clade A (bs = 100) although it forms a sister group to reference strains INGV and Mermet virus (Figure 1).

The phylogenetic analysis of a 503 nt sequence of the S segment (Figure 2) as well as the BLAST results against reference sequences confirmed the results obtained from the 152 nt sequence (Figure 1) for two samples as SHUV (KYA299_16, and MN055_16) and one sample as SBV (MAR538_16) (Figure 2). The phylogenetic analysis and BLAST of a 503 nt sequence of KYA233_16 identified the sample as SHUV, with this sample lacking a 152 nt sequence. For two samples, KYA358_17 and LAP066_13, conflicting phylogenetic- and p-distance results were seen. KAY358_17 has a 100% nucleotide identity to MAR538_16 (152 nt sequence of the S segment) that clustered within clade B, although it collapsed, probably due to incomplete sampling or lack of reference sequences. In contrast, based on the phylogenetic analysis (Figure 2) and BLAST results (Table S2) of the 503 nt sequence of the S segment, KAY358_17 groups with SHUV, and therefore was confidently identified as such. Sample LAP066_13 was recovered as sister to SHUV, PEAV and AINV, and MAR278_14 (sister to LAP066_13 + (SHUV + PEAV + AINV)), and therefore could not confidently be identified based on the 152 nt sequence of the S segment (Figure 1). LAP066_13 also clustered separately (bs = 100) to SHUV, PEAV, and AINV on the 503 nt sequence of the S segment (Figure 2) re-affirming aforementioned grouping (Figure 1), and therefore remains unknown.

**Figure 3:**
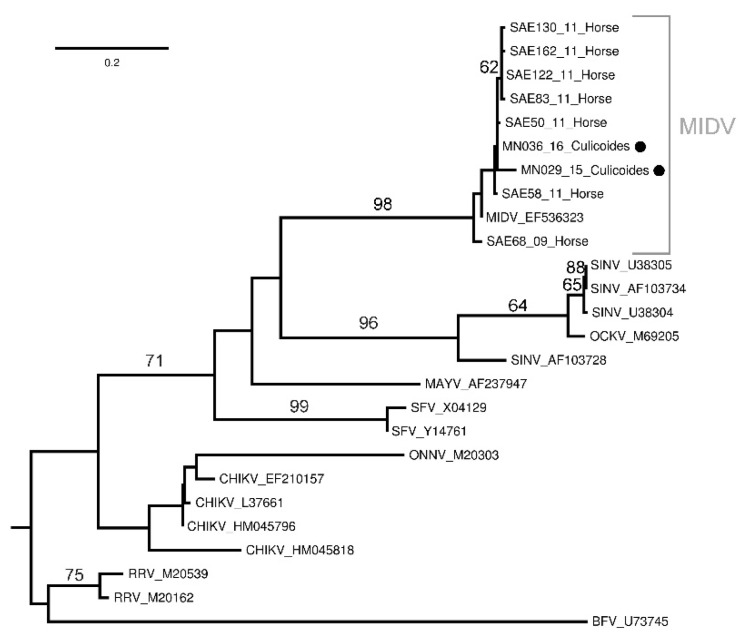
Maximum likelihood phylogram of the partial (nucleotides = 198, 26 taxa) *Alphavirus* NsP4 gene region. Bootstrap support calculated using autoMRE function invoked in RAxML (GTR + G model). Barmah Forest (BFV) and Ross River virus (RRV) were used as outgroups. Filled black circles indicate new sequences.

Phylogenetic analyses based on a 198 nt gene region of the partial Alphavirus NsP4 gene confirmed the two MIDV positives, with samples MN29_15 and MN36_16 clustering with samples obtained from horses in South Africa (bs = 98) (Figure 3) with the p-distance analysis demonstrating high nucleotide similarities (99–100%) (not shown).

**Figure 4:**
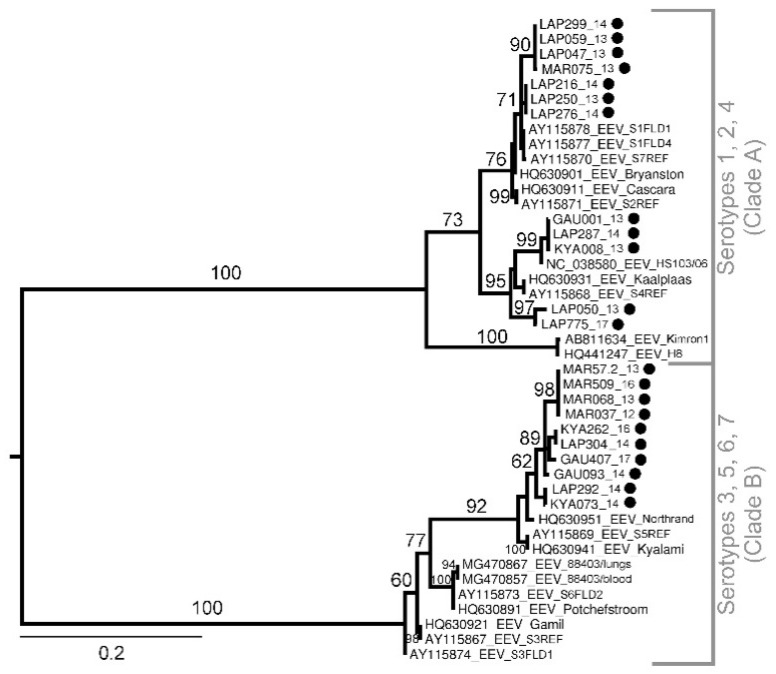
Maximum likelihood phylogram based on the partial (nucleotides = 401, 43 taxa) EEV NS3 segment. Bootstrap support calculated using autoMRE function invoked in RAxML (GTR + G model). Phylogram rooted at midpoint. Filled black circles indicate new sequences.

Equine encephalitis virus phylogenetic analysis based on the NS3 gene (Segment 10) (401 nt) (Figure 4) cluster sequences into two distinct (bs = 100) monophyletic groups, clade A and B. No geographical correlation is evident, as both the clades have included positive pools from all four sites and indicate circulation of various serotypes in these areas. On the basis of the partial NS3 gene, the majority of EEV positives cluster with serotypes 5 and 7 (Kyalami and Northrand).

**Figure 5:**
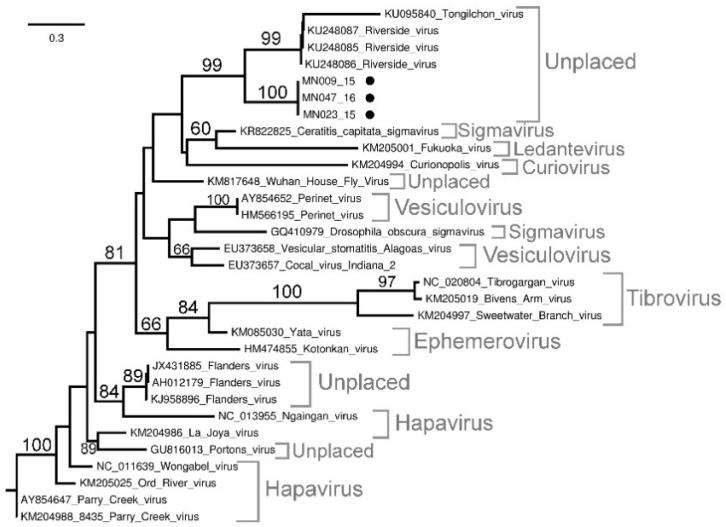
Maximum likelihood phylogram based on the partial (nucleotides = 310, 31 taxa) L segment of *Rhabdoviridae* viruses. Bootstrap support calculated using autoMRE function invoked in RAxML (GTR + G model). Filled black circles indicate new sequences.

Phylogenetic analysis of the L segment of three *Rhabdoviridae* viruses (Figure 5) confirmed the PCR results of MN009_15, MN23_15, and MN47_16, although the *Rhabdoviridae* positives remain unclassified. Samples MN009_15, MN047_16, and MN023_15 formed a well-supported sister group (bs = 100) to Riverside- (RISV) and Tongilchon virus (TCHV) (bs = 99) which, in turn, was well supported as a yet unclassified monophyletic group (bs = 99). The p-distance analysis (not shown) reveals the highest nucleotide identities (86.6%) of samples MN009_15, MN23_15, and MN47_16 to RISV (KU248086, KU248087, and KU248085, respectively).

## 4. Discussion

This study used field surveillance and molecular screening of *Culicoides* to detect viral RNA previously implicated in outbreaks in animals in South Africa and establish a possible association of *Culicoides* as vectors for the identified viruses. Midge pools, collected from 2012 to 2017, tested positive for viruses from the *Alphavirus* and *Orthobunyavirus* genera and family *Rhabdoviridae* as well as EEV. No *Flavivirus* or *Phlebovirus* positives were reported. The highest positivity of viruses was detected in February followed by January and March. This is typical seasonality for arboviruses in South Africa as the arbovirus season is late summer to autumn (February to May). Pools collected during winter months were small or not collected at all, resulting in no positives during July. Overwintering of viruses has, however, been demonstrated for BTV [26] and AHSV [27] in South Africa, even at low insect activity.

Orthobunyaviruses, specifically, SHUV had the highest prevalence across the sites with the highest minimum infection rate in Marakele National Park, while no orthbunyaviruses were detected in KNP in our collection. Phylogenetic analysis based on a 152 nt sequence of the S segment clustered the newly sequenced SHUV strains with known SHUV sequences from horses [28] possibly indicating transmission from midges. In addition, the strains that clearly grouped with SHUV, 15 other orthobunyviruses were identified as SHAV, SABV, SANV, SBV, SATV, and INGV, although the phylogenetic clustering of the Simbu serogroup could not confidently be resolved to the species level due to short-sequenced regions. The Shamonda, Sabo, and Sango viruses have previously been identified in parts of Africa, the Middle East, and Asia as a livestock disease responsible for fetal congenital deformities [29,30] and little is known on the zoonotic potential of these viruses. The Schmallenberg virus has been responsible for large outbreaks of abortion storms and fetal malformations in cattle resulting in large economic losses in Europe [3]. The Schmallenberg virus RNA was also recently detected in *Culicoides* pools as well as in malformed livestock in Israel [31]. Antibodies to SBV have been reported in ruminants of neighboring countries of Namibia [32] and Mozambique [33], indicating the virus may be circulating in southern Africa; however, the close relationship with other members of this group, such as is apparent from our study, may also suggest possible cross reactivity of antibodies in the Simbu serogroup [34]. The Ingwavuma virus was the only virus not from clade B in the Simbu serogroup. The Ingwavuma virus has been isolated in South Africa from a bird and two pools of *Culex* mosquitoes [35]. Since then, antibodies to INGV have been detected in water buffaloes, goats, sheep, and pigs in part of Indonesia and Thailand where pigs are natural hosts of INGV [36,37].

Additional PCR assays were successful in obtaining longer sequences for six orthobunyavirus positive samples and could confidently identify five viruses based on the S segment. One orthobunyavirus positive remained unidentified (LAP066_13). This may suggest a new or uncharacterized virus and requires further investigation including additional reference sequences. The conflicting data for sample KYA358_17 could be because the S segment is highly conserved, where the smaller sequence is very similar to various members of the broader Simbu complex with the larger, 503 nt sequence revealing higher identity to SHUV. The variability of the S segment between strains could also be due to reassortment as the multi-segmented nature of orthobunyaviruses allows for genetic exchange to increase the fitness, spread, and pathogenicity of the virus or serogroup [38]. The inability to generate larger sequences for all samples or sequence to the M and L gene segment could be due to the low level of RNA present in the vector pool or due to genetic differences in the primer area that was designed based on SHUV sequences isolated from horses.

During the six-year surveillance, MIDV was only detected twice in midge pools from KNP, despite detecting animal cases [13]. The low positivity of MIDV in *Culicoides* pools during the surveillance period suggests that the virus is present in the area and that the midges were able to ingest the virus but that they might play a less important role as a vector. Phylogenetic analyses cluster the MIDV detected in the midges with MIDV strains found in horses [18]. This could indicate circulation of various genotypes in that area or structural changes in the gene caused by the midge ingestion [39]. No flaviviruses, such as WNV, were detected in this study. Previous studies have reported on WNV detection in *Culicoides* [7], although their role in the epidemiology of WNV is still unclear.

The high prevalence of EEV is not surprising, as *Culicoides* midges, specifically *C. imicola* and *Culicoides bolitinos*, are the primary vectors for EEV. Cases of febrile, neurological, and respiratory disease in horses infected with EEV were also frequently detected across South Africa during the same period [14]. Equine encephalosis virus detected in midges formed two distinct clusters with the majority grouping with the Kyalami and Northrand serotypes (clade B) and not the Bryanston and Potchefstroom serotypes, as previously reported [40]. No EEV was detected in midge pools from Mnisi or KNP with the highest EEV MIR reported in Lapalala Wilderness. Previous studies have indicated antibodies to EEV in zebras (*Zebra burchelli*) from KNP [41], although less is published on the epidemiology of EEV and the circulation of this predominantly horse pathogen within wildlife areas. Further investigations into the role of wildlife reservoirs are needed.

The four viruses from the *Rhabdoviridae* family were accidentally discovered after the flavivirus genus PCR produced bands of the right size on gel electrophoreses. Sanger sequence and p-distance analyses revealed uncharacterized viruses from the *Rhabdoviridae* family. The phylogenetic analysis of the L segment clusters these viruses with unclassified viruses (RISV and TCHV), although further sequence data is needed to characterize the virus species. The Riverside virus was first isolated in Hungary from an unknown *Aedes* species, while TCHV was isolated in South Korea from *Culex bitaeniorhynchus* [42,43]. Grouping of MN009_15, MN23_15, and MN47_16 with mosquito-associated viruses rather than with *Culicoides*-associated viruses such as Sweetwater Branch, Bivens Arm, and Tibrogargan virus indicates possible incidental ingestion. Another reason could be that both RISV and TCHV were only recently discovered and could be associated with *Culicoides* and warrant further investigation. The *Culicoides*-transmitted *Rhabdoviridae* viruses has been implicated as disease-causing agents in Australia, the Americas, Asia, and Africa [44,45], as well as bovine ephemeral fever virus in South Africa [39].

Unfortunately, virus isolation was unsuccessful on mammalian cells (Vero and BHK) and will, in the future, be attempted on insect cells such as C6/36 or KC cell. Further attempts at next generation sequencing and virus isolation will be made to resolve the unplaced and unidentified viruses. A limiting factor could be large pool sizes resulting in decreasing detection rate, while future studies could benefit by decreasing pool sizes to <100.

The six vector surveillance sites were selected based on previous outbreaks of neurological disease in animals due to arbovirus infections as well as the strong humans–animal interface at these sites [10,11,12,13,14,15]. The highest overall MIR was reported in Lapalala Wilderness. Studies have indicated a MIR ≥1 result in outbreaks of arboviruses such as WNV and eastern equine encephalitis virus in the Americas [46,47]. Although the *Orthobunyavirus* MIR in Lapalala Wilderness was <1, indicating a low probability of an outbreak, a SHUV outbreak in wildlife occurred from the end of 2017 to mid-2018 [12]; only a small portion of the *Culicoides* population was sampled and more extensive surveillance could have accurately predicted the outbreak. Marakele National Park had the highest virus diversity with various orthobunyaviruses and EEV detected. In Mpumalanga, there is a strong human–livestock–wildlife interface at the collection sites. Spillovers of disease from wildlife to domestic animals have been reported in these areas [48]. In Boschkop and Kyalami, numerous outbreaks of WNV [10], SHUV [27], MIDV [18], and EEV [14] in horses have been reported, which consist of agricultural small holdings rather than wildlife areas. Morphological identification of *Culicoides* species from subsamples collected at the various surveillance sites demonstrates the high abundance of *C. imicola* at all sites, supporting previous findings [49,50]. The abundance of *C. imicola* and *Culicoides schultzei* in areas with high infection rates could provide valuable insight into these species as vectors for emerging arboviruses in South Africa.

Taking everything into consideration, detection of viral RNA by PCR does not prove vector status for *Culicoides* midges, because oral susceptibility and transmission as well as the association of the infected arthropod with the vertebrate population in which the infection is occurring need to be demonstrated [1]. Screening of field collected *Culicoides*, however, represent a non-invasive method to determine virus presence in an area without using invasive methods such as to immobilize and bleed animals or involved human patients. Previous studies have also implicated field collected *Culicoides* in the spread of SHUV [8] and successful virus dissemination has been demonstrated for SHUV in the European *Culicoides nubeculosus* and the North American *Culicoides sonorensis* [51].

This study identified viruses of medical and veterinary importance from RNA isolated from *Culicoides* collected in wildlife and urban areas in South Africa. Even though vector competence was not shown, the data suggest that *Culicoides* may play a role in disease outbreaks warranting further investigations into their potential as vectors for the identified viruses and their capacity to spread these pathogens to new regions.

## Figures and Tables

**Table 1:** *Culicoides* collection site information located in three provinces in South Africa, number (no.) of *Culicoides* collected per site and average catch size ($\bar{x}$) from 2012 to 2017. Adapted from (5).

Site	Location Code	Province	Animals Near Light Trap	Sampling Period	Trap Nights	No. of *Culicoides* Collected	Average Catch Size ($\bar{x}$)
Hluvukani (Mnisi)/Hlalakahle/Ludlow (Mnisi)	MN	Mpumalanga	Humans, dogs, pigs, goats, cattle, donkeys	2015/2016–2017	45	37,612	836
Manyeleti/HHWRS (KNP)	MN/KNP		Wildlife, humans	2015/2016–2017	25	14,519	580
Lapalala Wilderness	LAP	Limpopo	Wildlife, humans	November 2012–2017	142	40,555	285
Marakele Natural Park	MAR		Wildlife, humans	September 2012–2017	157	89,087	567
Boschkop	GAU	Gauteng	Horse, human, goats and sheep	2013–2017	88	107,863	1225
Kyalami	KYA		Horse, humans, goats and sheep	2013–2017	75	1,953,680	25,782
Total					532	222,329	4179

HHWRS, Hans Hoheisen Wildlife Research Station; KNP, Kruger National Park.

**Table 2:** Viruses detected at the six collection sites, in three provinces in South Africa, with total number (no.) of *Culicoides* pools screened by PCR, number of positives pools for each viruses detected as well as the minimum infection rate (MIR) with 95% confidence interval (CI) indicated. *Culicoides* were collected monthly from September 2012 to May 2017 for two consecutive nights using 220 V Onderstepoort light traps.

					MIR 95% CI			
Site	No. Pools Tested	No. Pools Positive	All Positives	SHUV	Other Orthos	MIDV	EEV	Rhabdo
Mnisi	47	10	0.4 (0.2–0.7)	0.2 (0.0–0.3)	0.1 (0.0–0.2)	-		0.2 (0.0–0.3)
KNP	27	2	0.2 (0.0–0.4)	-	-	0.1 (0.0–0.4)		-
Boschkop	64	9	0.3 (0.1–0.5)	0.0 (0.0–0.1)	0.2 (0.0–0.3)	-	0.1 (0.0–0.2)	-
Kyalami	59	9	0.3 (0.1–0.5)	0.1 (0.0–0.3)	0.1 (0.0–0.2)	-	0.1 (0.0–0.2)	-
Lapalala	49	20	0.8 (0.5–1.2)	0.1 (0.0–0.3)	0.0 (0.0–01)	-	0.7 (0.3–1.0)	-
Marakele	85	19	0.5 (0.2–0.6)	0.1 (0.0–0.3)	0.1 (0.0–0.2)	-	0.2 (0.1–0.3)	-
Total	331	69	0.4 (0.3–0.5)	0.1 (0.1–0.2)	0.1 (0.0–0.1)	0.01 (0.0–0.03)	0.2 (0.1–0.2)	0.02 (0.0–0.05)

Mnisi, MN; Kruger National Park, KNP; Kyalami, KYA; Boschkop, GAU; Marakele, MAR; Lapalala, LAP; Shuni virus, SHUV; Middelburg virus, MIDV; equine encephalosis virus, EEV; *Orthobunyaviruses,* Orthos; *Rhabdoviridae*, Rhabdo

## Data Availability

The data that support the findings of this study are openly available from GenBank, accession numbers MN270991–MN271022.

## Supplementary Materials

The following are available online at https://www.mdpi.com/article/10.3390/v13101978/s1: Table S1, *Culicoides* species morphologically identified from subsamples collected at six vector surveillance sites in South Africa from 2012–2017 using 220 V Onderstpoort Light traps. Adapted from [3], Table S2, Collection information pertaining to the *Culicoides* pools screened in this study as well as phylogenetic and BLAST results of the viruses (family and species) detected in midge pools. GenBank accession number are indicated where applicable.

## Appendix A

For one sample, MN055-16, a partial (395 nt) M segment, was obtained (accession number in Table S2). The SHUV M segment primers were designed based on the genomic position of reference SHUV sequences from GenBank. The conserved region between amino acid positions 2306 and 3266 of the M segment was used to identify first round (forward 23065′-AGCATGYGCYAGGGATACAC-3′2326 and reverse 32665′-CTGRCTGATTAATGGCTGC-3′3247) and nested (forward 23415′-TTGAACCAGCAACACGAGGA-3′2361 and reverse 27595′-ATGTGGGTGCAATRGARGGC-3′2739) primers using the NCBI Primer designing tool (National Center for Biotechnology Information, www.ncbi.nlm.nih.gov (accessed on 30 March 2018)) resulting in a nested PCR product of 418 bp. The cDNA was synthesised using a RevertAid First Strand cDNA Synthesis Kit (Thermo Fisher Scientific, Waltham, MA, USA) and random priming (Sigma-Aldrich, St. Louis, Mo, USA) according to the manufacturer’s instructions, incubated for 5 min at 25 °C, then 60 min at 45 °C, and finally for 5 min at 70 °C. The resulting cDNA was used in a first round PCR using Platinum™ Taq DNA polymerase (Thermo Fisher Scientific). Each 25 µL contained 10 pmol forward and reverse primers, Platinum™ Taq DNA polymerase buffer, 50 mM MgCl_2_ (Thermo Fisher Scientific), 10 mM dNTPs (Thermo Fisher Scientific), and Platinum™ Taq DNA Polymerase enzyme. Cycling conditions were as follow: 94 °C for 2 min, followed by 35 cycles of 94 °C for 30 s, 55 °C for 1 min, and 72 °C for 1 min, and a final extension of 72 °C for 7 min. A nested PCR was, then, also performed using Platinum™ Taq DNA polymerase (Thermo Fisher Scientific). Each 25 µL contained 10 pmol nested forward and nested reverse primers, Platinum™ Taq DNA polymerase buffer, 50 mM MgCl_2_ (Thermo Fisher Scientific), 10 mM dNTPs (Thermo Fisher Scientific), and Platinum™ Taq DNA polymerase enzyme. The cycling conditions were the same as the first round PCR.
