# Supplementary material for: An Investigation of Culicoides (Diptera: Ceratopogonidae) as Potential Vectors of Medically and Veterinary Important Arboviruses in South Africa
Source: Viruses. 2021 Oct 1;13(10):1978. doi: 10.3390/v13101978 (PMC8541229; doi:10.3390/v13101978)
Supplement: Supplementary file 1 [file viruses-13-01978-s001.zip › viruses-1364919-supplementary.pdf]

Supplementary Material

Table S1: *Culicoides* species morphologically identified from sub samples collected at six vector surveillance sites in South Africa from 2012-2017 using 220V Onderstspoort Light traps. Adapted from [5].

Culicoides species	Kruger National Park	Mnisi	Marakele National Park	Lapalala	Kyalami	Gauteng	Total
C. albopunctatus	3						3
C. bolitinos	9		1			2	12
C. enderleini	7	2	2				11
C. eriodendroni	2						2
C. exspectator	27	8					35
C. imcola	49	28	20	12	45	43	197
C. leucosticus	42	7	3	2		1	55
C. loxodontis	2						2
C. neavei		1					1
C. nevilli		1					1
C. nigeriae	3						3
C. nigripennis grp	2						2
C. nivosus	1	5	2				8
C. olyslageri	2						2
C. pretoriensis	4						4
C. punctithorax	2						2
C. pycnosticus	12	1	6				19
C. ravus	11	7	1				19
C. schultzei			11				11
C. similis	4		1				5
C. sp. # 61	1						1
C. subschultzei	6	7					13
C. tropicalis		3					3
C. tuttifrutti		6					6
C. walkeri	1						1
Total	190	76	47	14	45	46	418

Table S2: Collection information pertaining to the *Culicoides* pools screened in this study as well as phylogenetic and BLAST results, of the viruses (family and species) detected in midge pools. GenBank accession number are indicated where applicable.

Pool ID	Site	Collection date	Family	Species	Sequence length (nt)	Accession number
MAR 403-15	Marakele	2015 May	*Peribunyaviridae*	INGV	152	NA
MAR 278-14	Marakele	2014 Apr	*Peribunyaviridae*	PEAV	152	NA
LAP 066-13	Lapalala	2013 Feb	*Peribunyaviridae*	Unknown	711	MN270995
GAU 093-14	Boschkop	2014 Feb	*Peribunyaviridae*	SABV	152	NA
MAR 085-13	Marakele	2013 Mar	*Peribunyaviridae*	SABV	152	NA
KYA 333-16	Kyalami	2016 Nov	*Peribunyaviridae*	SABV	152	NA
KYA 352-17	Kyalami	2017 Jan	*Peribunyaviridae*	SABV	152	NA
MAR 032-12	Marakele	2012 Nov	*Peribunyaviridae*	SABV	152	NA
MN 040-16	Mnisi	2016 Feb	*Peribunyaviridae*	SATV	152	NA
MAR 538-16	Marakele	2016 May	*Peribunyaviridae*	SBV	766	MN270994
GAU 110-14	Boschkop	2014 Apr	*Peribunyaviridae*	SHAV	152	NA
GAU 272-16	Boschkop	2016 Apr	*Peribunyaviridae*	SHAV	152	NA
GAU 290-16	Boschkop	2016 Jun	*Peribunyaviridae*	SHAV	152	NA
GAU 372-17	Boschkop	2017 Jan	*Peribunyaviridae*	SHAV	152	NA
MN 048-16	Mnisi	2016 Mar	*Peribunyaviridae*	SHAV	152	NA
GAU 388-17	Boschkop	2017 Mar	*Peribunyaviridae*	SHUV	152	NA
KYA 077-14	Kyalami	2014 Feb	*Peribunyaviridae*	SHUV	152	NA
KYA 229-16	Kyalami	2016 Feb	*Peribunyaviridae*	SHUV	818	MN270996
KYA 233-16	Kyalami	2016 Mar	*Peribunyaviridae*	SHUV	825	MN270997

KYA 358-17	Kyalami	2017 Jan	*Peribunyaviridae*	SHUV	538	MN270998
LAP 152-13	Lapalala	2013 Sep	*Peribunyaviridae*	SHUV	152	NA
LAP 341-14	Lapalala	2014 Jun	*Peribunyaviridae*	SHUV	152	NA
LAP 775-17	Lapalala	2017 Feb	*Peribunyaviridae*	SHUV	152	NA
MAR 057.2-13	Marakele	2013 Feb	*Peribunyaviridae*	SHUV	152	NA
MAR 062-13	Marakele	2013 Feb	*Peribunyaviridae*	SHUV	152	NA
MAR 178-13	Marakele	2013 Oct	*Peribunyaviridae*	SHUV	152	NA
MAR 205-14	Marakele	2014 Jan	*Peribunyaviridae*	SHUV	152	NA
MAR 206-14	Marakele	2014 Jan	*Peribunyaviridae*	SHUV	152	NA
MAR 395-15	Marakele	2015 Feb	*Peribunyaviridae*	SHUV	152	NA
MN 055-16	Mnisi	2016 Apr	*Peribunyaviridae*	SHUV	434<br>395	MN270999 (S)<br>MN271000 (M)
MN 043-16	Mnisi	2016 Feb	*Peribunyaviridae*	SHUV	152	NA
MN 045-16	Mnisi	2016 Feb	*Peribunyaviridae*	SHUV	152	NA
KRU 139-16	Mnisi	2016 Nov	*Peribunyaviridae*	SHUV	152	NA
GAU 001-13	Boschkop	2013 Mar	*Reoviridae*	EEV	401	MN271011
GAU 093-14	Boschkop	2014 Feb	*Reoviridae*	EEV	401	MN271003
GAU 407-17	Boschkop	2017 Apr	*Reoviridae*	EEV	401	MN271008
KYA 008-13	Kyalami	2013 Feb	*Reoviridae*	EEV	401	MN271012
KYA 073-14	Kyalami	2014 Feb	*Reoviridae*	EEV	401	MN271009
KYA 262-16	Kyalami	2016 May	*Reoviridae*	EEV	401	MN271001
LAP 059-13	Lapalala	2013 Feb	*Reoviridae*	EEV	401	MN271017
LAP 047-13	Lapalala	2013 Jan	*Reoviridae*	EEV	401	MN271016

LAP 050-13	Lapalala	2013 Jan	*Reoviridae*	EEV	401	MN271015
LAP 299-14	Lapalala	2014 Apr	*Reoviridae*	EEV	401	MN271018
LAP 304-14	Lapalala	2014 Apr	*Reoviridae*	EEV	401	MN271002
LAP 250-14	Lapalala	2014 Feb	*Reoviridae*	EEV	401	MN271022
LAP 216-14	Lapalala	2014 Jan	*Reoviridae*	EEV	401	MN271020
LAP 276-14	Lapalala	2014 Mar	*Reoviridae*	EEV	401	MN271021
LAP 287-14	Lapalala	2014 Mar	*Reoviridae*	EEV	401	MN271013
LAP 288-14	Lapalala	2014 Mar	*Reoviridae*	EEV	No sequence data	NA
LAP 292-14	Lapalala	2014 Mar	*Reoviridae*	EEV	401	MN271010
LAP 314-14	Lapalala	2014 May	*Reoviridae*	EEV	No sequence data	NA
LAP 531-15	Lapalala	2015 Oct	*Reoviridae*	EEV	No sequence data	NA
LAP 588-16	Lapalala	2016 Feb	*Reoviridae*	EEV	No sequence data	NA
LAP 601-16	Lapalala	2016 Marc	*Reoviridae*	EEV	No sequence data	NA
LAP 775-17	Lapalala	2017 Feb	*Reoviridae*	EEV	401	MN271014
MAR 037-12	Marakele	2012 Dec	*Reoviridae*	EEV	401	MN271004
MAR 057.2-13	Marakele	2013 Feb	*Reoviridae*	EEV	401	MN271005
MAR 058.2-13	Marakele	2013 Feb	*Reoviridae*	EEV	No sequence data	NA
MAR 068-13	Marakele	2013 Feb	*Reoviridae*	EEV	401	MN271006
MAR 075-13	Marakele	2013 Mar	*Reoviridae*	EEV	401	MN271019

MAR 289-14	Marakele	2014 Apr	*Reoviridae*	EEV	No sequence data	NA
MAR 304-14	Marakele	2014 May	*Reoviridae*	EEV	No sequence data	NA
MAR 509-16	Marakele	2016 Feb	*Reoviridae*	EEV	401	MN271007
MN 014-15	Mnisi	2015 Aug	*Rhabdoviridae*	Rhabdo viridae	*No sequence data	NA
MN 009-15	Mnisi	2015 Jun	*Rhabdoviridae*	Rhabdo viridae	310	MN270991
MN 023-15	Mnisi	2015 Nov	*Rhabdoviridae*	Rhabdo viridae	310	MN270993
MN 047-16	Mnisi	2016 Mar	*Rhabdoviridae*	Rhabdo viridae	310	MN270992
MN 029-15	KNP	2015 Nov	*Togaviridae*	MIDV	198	NA
MN 036-16	KNP	2016 Dec	*Togaviridae*	MIDV	198	NA

SHUV: Shuni Virus; PEAV: Peaton; SHAV: Shamonda virus, SABV: Sabo virus, SANV: Sango virus, SBV: Schmallenberg virus, SATV: Satuperi virus; INGV: Ingwavuma virus; EEV: Equine encephalitis virus. No sequence data: Detection of virus by EEV-specific FAM-TaqMan® probe or *incomplete sequence.
